# Resource reallocation under persistent immune activation drives trade-offs between life history and immunity in pirk-deficient *Musca domestica*

**DOI:** 10.1186/s12915-025-02324-6

**Published:** 2025-07-22

**Authors:** Ting Tang, Lan Yang, Liya Ma, Yu Ren, Mengnan Li, Shufan Guo, Xin Wang, Yuming Zhang, Fengsong Liu

**Affiliations:** 1 The Key Laboratory of Zoological Systematics and Application, College of Life Sciences, Hebei University, Baoding, 071002 China; 2 Hebei Basic Science Center for Biotic Interaction, Hebei University, Baoding, 071002 China

**Keywords:** *Musca domestica*, Immune regulation, Energy metabolism, Trade-off, *Pirk*

## Abstract

**Background:**

The activation of the immune system by pathogens imposes significant energetic costs on hosts, which may result in the diversion of resources away from other non-essential biological processes, such as growth and reproduction. The underlying mechanisms of trade-offs between immune responses and host fitness remain poorly understood.

**Results:**

We used a *Musca domestica* mutant (pirk-KO) to evaluate the influence of non-infection-induced immune system activation on female reproduction and larval growth. Pirk, a negative feedback inhibitor of the immune deficiency (Imd) pathway expressed in intestine and fat body, was induced by bacteria. *pirk* loss enhanced the immune response of house flies, reflected in sustained upregulated antimicrobial peptide gene expression and improved resistance to bacterial infections. The phenotypic traits of pirk-KO house flies, including delayed larval growth, reduced the body weight, and impaired female fertility, were indicative of the adaptive costs associated with aberrant immune activation. The transcriptional heterogeneities between pirk-KO and wild-type (WT) male flies indicated the overactivation of the Imd signaling pathway, accompanied by significant metabolic adaptations to the loss of pirk. The upregulation of pivotal genes involved in glycolysis and the TCA cycle indicated an enhanced central carbon metabolism in pirk-KO. The downregulation of multiple key enzymes involved in the pentose phosphate pathway in pirk-KO flies suggests a reduction in metabolic flux through the pentose phosphate pathway, which in turn results in impaired anabolism. The collective findings indicate that the pirk-KO flies undergo metabolic reprogramming to increase ATP production as a response to excessive immune activation, rather than incorporating nutrients into cellular biomass for cell proliferation. The pirk-KO flies exhibited a significantly elevated food intake and elevated levels of free glucose, trehalose, and fructose in comparison to the WT flies. Nevertheless, the glycogen and triglyceride contents in the pirk-KO flies were observed to be slightly diminished in comparison to the WT group.

**Conclusions:**

When the immune defense is activated, the flies extract more free energy to fuel the immunological deployment by increasing nutrient intake, as well as reducing resource allocation to non-essential life-history traits, primarily reproduction and growth.

**Supplementary Information:**

The online version contains supplementary material available at 10.1186/s12915-025-02324-6.

## Background

Hosts are subjected to persistent assaults from pathogens in their natural habitats, which exert considerable selective pressure on their immune systems, driving rapid immune evolution [1]. Insects have a highly developed innate immune system that enables them to effectively defend themselves against pathogens. This renders insects an invaluable model for the study of immune system evolution. Recent research in the fruit fly *Drosophila melanogaster* has revealed the influence of ecology on the evolution of the immune system and provided insights into the mechanisms underlying host defense against pathogen infection. Unlike mammals, insects lack an adaptive immune response and instead rely on innate immunity, which encompasses both humoral and cellular defenses to resist invading pathogens [2–4]. The humoral immune response is typified by the synthesis of antimicrobial peptides (AMPs) from the fat body and hemocytes, which are regulated by a few signaling pathways, including Toll signaling pathway, immune deficiency (Imd) pathway, c-Jun N-terminal kinase (JNK) pathway, and Janus kinase/signal transducer and activator of transcription (JAK/STAT) pathway. In contrast, the cellular immune response employs hemocytes to immobilize pathogens through phagocytosis, encapsulation, and melanization.

Although the immune response is essential for survival, it has been proposed that it may be energetically expensive and may reduce host fitness by diverting resources from other life-history traits, such as growth and reproduction [5–7]. Life-history theory posits that physiological trade-offs may influence the allocation of resources among costly life-history traits [8]. Over the past two decades, there has been a significant body of research examining the trade-offs between traits related to fitness, such as fecundity versus survival and fecundity versus immunity [6, 9–13]. Nevertheless, the regulatory mechanism responsible for these trade-offs across different traits remains unclear. To prevent excessive energy expenditure and autoimmune damage, insects have evolved a variety of negative regulatory mechanisms that suppress immune activation in the absence of infection or hyperactivation upon infection at multiple levels [14–16]. Basal negative regulators, including PGRP-LF, DNR, dUSP36, POSH, CYLD, SKPA/SLMB/DCUL1, Caspar, Caudal, and Trabid, serve to prevent the constitutive activation of the Imd pathway in *Drosophila* [17, 18]. Inducible negative regulators, including PGRP-SC, PGRP-LB, and pirk, modulate the magnitude of the Imd response. The *pirk* gene (poor Imd response upon knock-in) is markedly induced in *Drosophila* following Gram-negative bacterial infection. Overexpression of Pirk has been observed to suppress the Imd pathway response and increase the host’s susceptibility to bacterial infection. Mechanistically, Pirk inhibits the activation of the Imd pathway by interfering with the interaction between PGRP-LC and -LE and the molecule Imd [15].

The *Musca domestica*, commonly known as the house fly, represents a promising subject for the study of innate immunity due to its immune system is not only evolutionarily conserved but also uniquely adapted to its septic ecological niche. Transcriptomic analyses of *M. domestica* have highlighted the rapid induction of immune-related genes, such as pattern recognition receptors (PRRs), the Toll and Imd pathways, and AMPs. Notably, *M. domestica* shows significant gene family expansions in immunity-related genes compared to *D. melanogaster*, particularly in families such as PGRPs, fibrinogen, thioester-containing protein, AMPs, and serpins [19, 20]. These expansions likely reflect functional redundancy, which may be associated with the need for a more rapid immune response to environmental changes. These differences suggest that *M. domestica*, under long-term exposure to decomposing organic material and pathogens, has evolved a more complex and resilient immune defense system through gene family expansions, regulatory network optimization, and the coordination of multiple systems, providing a defense mechanism that surpasses that of *D. melanogaster*. In the present study, the *M. domestica* genome was found to contain a *pirk* gene, which was subsequently demonstrated to play a role in the negative regulation of antibacterial defense. A house fly *pirk* mutant was generated using CRISPR/Cas9, thereby providing an invertebrate model for the study of the fitness cost associated with immune responses in the absence of pathogen interference.

## Results

### Identification of the *pirk* gene in *M. domestica*

By screening the genomic and transcriptomic data of *M. domestica*, an ortholog of *D. melanogaster pirk* was identified and designated as *M. domestica pirk*. The cDNA sequence containing the complete open reading frame of *pirk* (GenBank accession number: XM_005188521.4) was amplified by RT-PCR and sequenced by the Sanger method. A sequence analysis revealed that *pirk* contains an open reading frame of 897 base pairs (bp), encoding 298 amino acid residues. The predicted molecular weight of the *M. domestica* Pirk protein is 32.8 kDa, and the predicted pI value is 9.23.

**Fig. 1:**
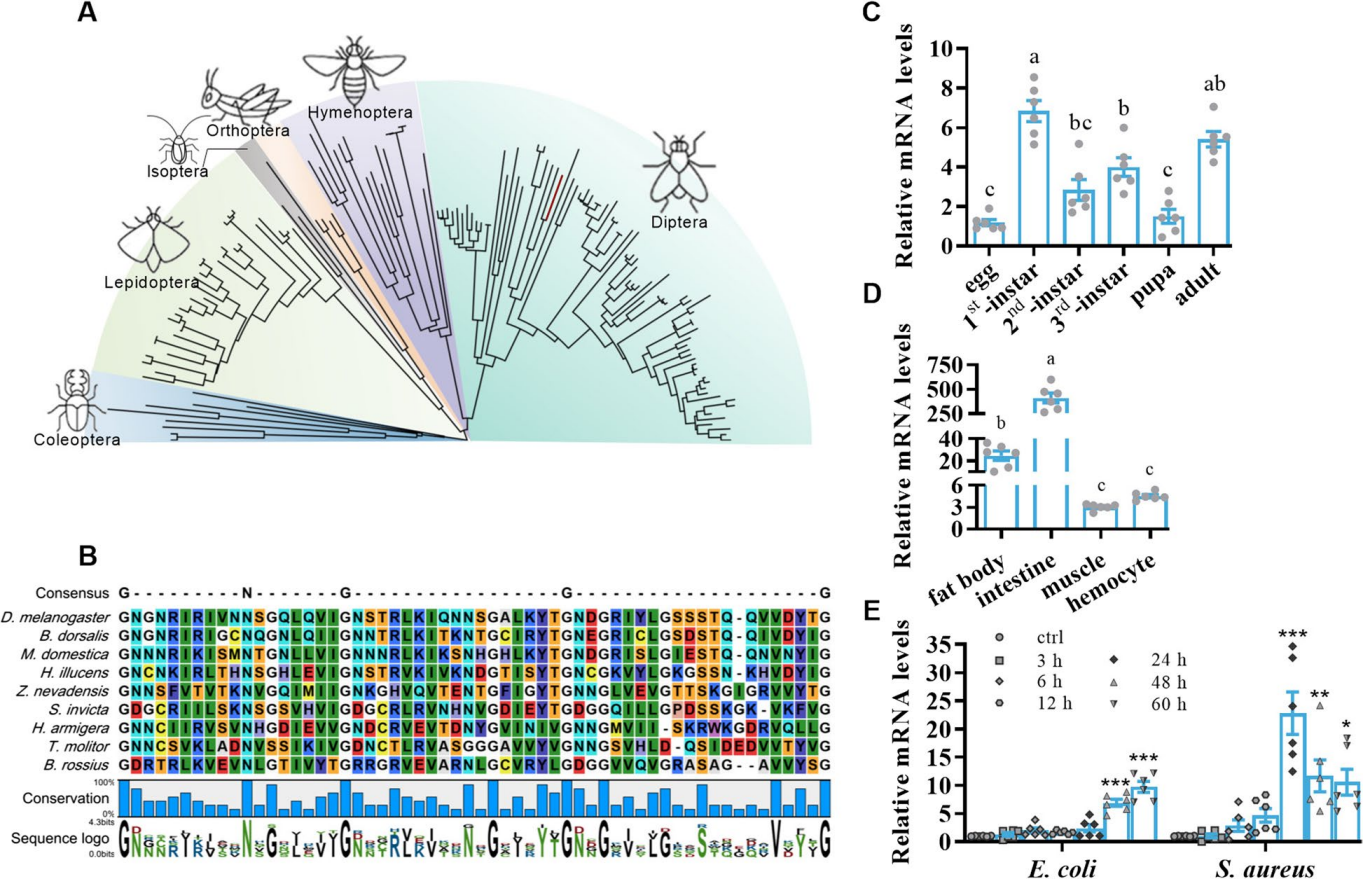
Bioinformatics and the transcript level analysis of *pirk* genes. **A** Phylogenetic tree comprising 147 Pirk protein sequences derived from 147 species across 7 orders. The maximum-likelihood method was employed to construct the phylogenetic tree using MEGA. The branches of the house fly are marked in red. Please refer to the SI section for the respective gene IDs and protein sequences analyzed in this study (SI-1). **B** Multiple sequence alignments of the Pirks. A representative from each of the orders was selected for the purpose of sequence alignment. The conservation of glycine and asparagine residues is demonstrated. **C** and **D** Expression of *pirk* was evaluated in different developmental stages and varied tissues of house fly through qRT-PCR analysis (*n* = 6, one-way ANOVA, Sidak’s and Tukey’s test, respectively). **E** The expression of *pirk* over time after challenge with *E. coli* or *S. aureus* was examined by qRT-PCR. The untreated samples from the 0 h time point was designated as the control group (ctrl) (*n* = 6, Brown-Forsythe and Welch ANOVA tests, Dunnett’s test). The error bar represents the mean ± SEM of results calculated based on three independent repeats. Statistical significance was indicated by different letters (*p* < 0.05). An asterisk indicates a significant difference from the control (*, *p* < 0.05; **, *p* < 0.01; ***, *p* < 0.001)

A BLAST search of the genome databases in GenBank identified a total of 147 Pirks homologous genes in 147 species (Additional file 1: SI-1). A comprehensive study of the 147 Pirks in a range of species from seven different orders including Coleoptera (9 species), Lepidoptera (33 species), Phasmatodea (1 species), Isoptera (2 species), Orthoptera (4 species), Hymenoptera (18 species), and Diptera (80 species) showed that these Pirks can be classified into six discrete groups (Fig. 1A). Multiple sequence alignment of the Pirks revealed the consensus pattern G-X_9_-N-X_7_-G-X_17_-G-X_20_-G, where X represents any amino acid residue (Fig. 1B). The results of the complete sequence alignment of Pirk proteins from 147 different species are available in Additional file 1: SI-2. A BLASTp analysis revealed that *M. domestica* Pirk shares 61.73%, 43.94%, and 26.86% identity with the homologous protein from *Musca vetustissima* (XP_061397944.1), *Stomoxys calcitrans* (XP_013117266.1), and *D. melanogaster* (NP_611598.1), respectively.

The results of the quantitative real-time polymerase chain reaction (qRT-PCR) demonstrated the expression of *M. domestica pirk* was examined at different developmental stages, and the findings indicated that *pirk* was expressed at comparable levels in larvae and adult flies, and at relatively low levels in eggs and pupae (see Fig. 1C). The expression of *pirk* in all examined tissues, with particularly high expression levels observed in the intestine and fat body, which were approximately 100- and sixfold higher, respectively, in comparison to hemocytes (Fig. 1D). The *pirk* expression profile was examined following challenge with either *Escherichia coli* or *Staphylococcus aureus*. In comparison to the control group, a notable elevation in *pirk* transcript levels was discerned during the course of infection, with the most pronounced expression occurring at 24 and 60 h following the challenge with *S. aureus* and *E. coli*, respectively (Fig. 1E).

### Generation of the *pirk*-knockout mutant in *M. domestica*

**Fig. 2:**
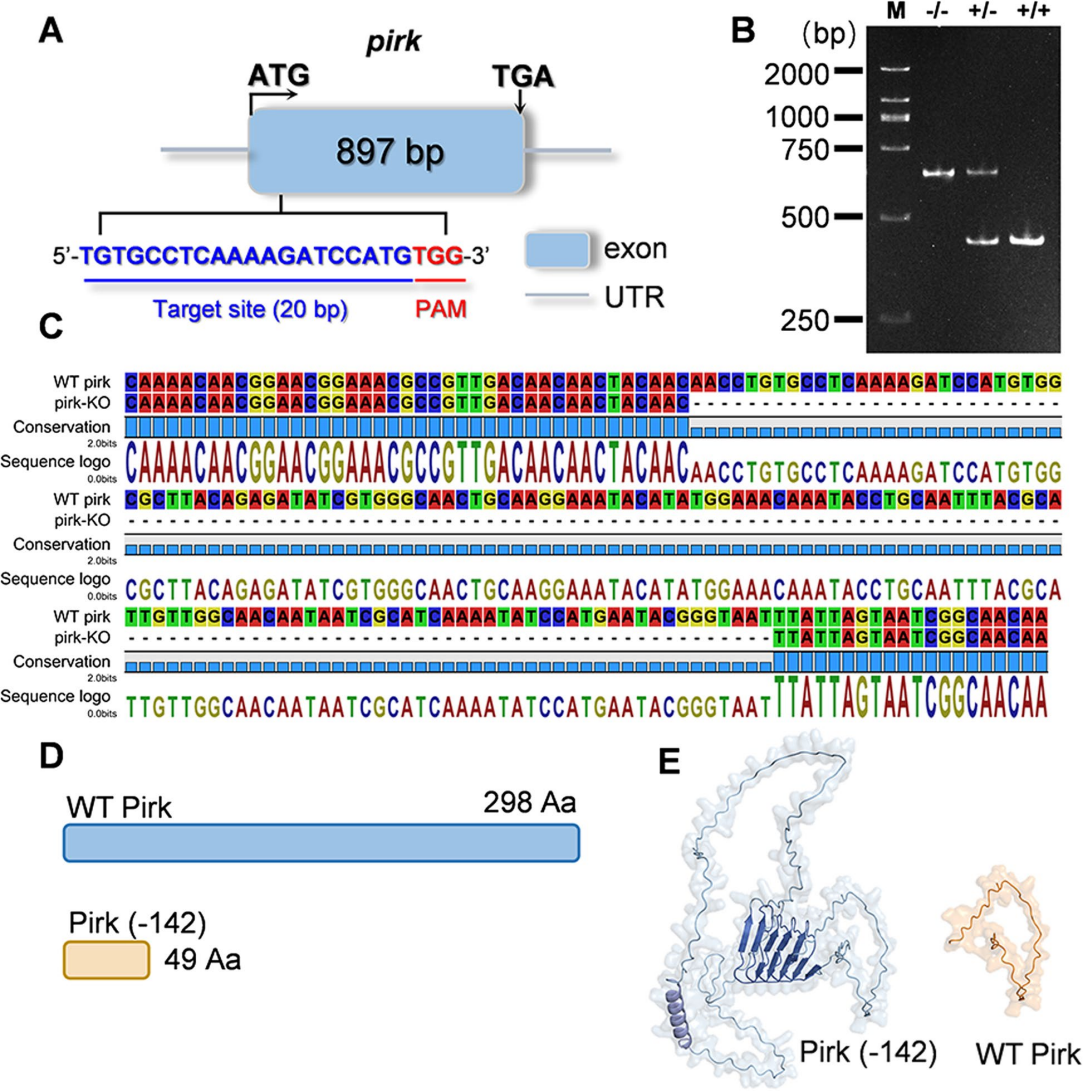
CRISPR/Cas9-directed heritable mutagenesis of *pirk* gene in *M. domestica*. **A** Schematic representation of the *pirk* gene structure. A gRNA targeting the exon sequence is indicated (PAM: red). **B** The homozygous mutant was detected using a 6% DNA-PAGE gel. The PCR product derived from the heterozygote (+ / −) contained two bands, whereas the WT (+ / +) and homozygous (− / −) had one band each, which could be easily distinguished on an acrylamide:bis (29:1) gel. The gel was electrophoresed at 150 V for 2.5 h in 1 × TBE buffer. Lane M: DNA marker. **C**, **D** The sequence alignment results following DNA sequencing revealed that a 142-bp deletion in the *pirk* gene in the mutant flies (compared to WT) results in premature translation termination. **E** Predicted protein structures of WT vs. 142-bp deletion pirk-KO alleles showing conformational differences

To investigate the immune functions of Pirk, we generated *pirk*-knockout house flies (pirk-KO) using the CRISPR technique. The *pirk* gene consists of a single exon of 897 bp. A guide RNA target was selected from the 5′ end of the exon (Fig. 2A). A mixture of gRNA and Cas9 protein was co-injected into 200 fresh eggs. The G0 generation of flies was then bred with wild-type (WT) individuals to produce the G1 generation. To identify the targeted mutations, genomic DNA was extracted from one of the hind legs of each G1 adult, and the resulting DNA was verified by means of both PCR and DNA sequencing analysis (Fig. 2B and C). One of the mutations, which exhibited a 142-bp deletion at exon, was inferred to produce a nonfunctional, truncated *M. domestica* Pirk protein (Fig. 2D and E). This mutation was selected for future self-crossing in order to achieve homozygotes.

### Growth arrest and impaired fertility in pirk-KO flies

**Fig. 3:**
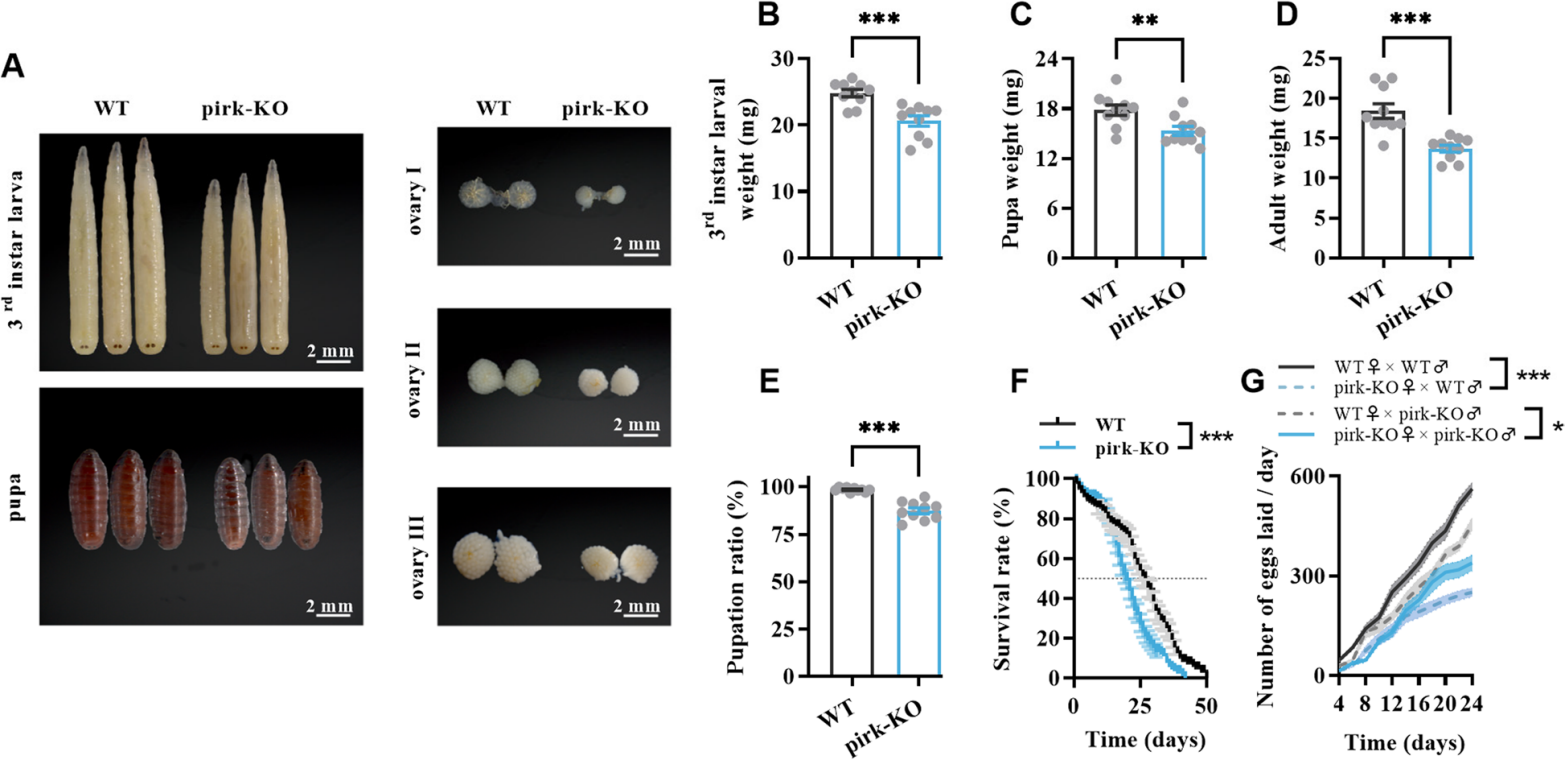
The loss of *pirk* function led to developmental and reproductive abnormalities. **A** The larvae, pupae, and ovaries were arranged to compare their sizes between the WT strain and *pirk* mutant under normal growth conditions at 28 °C. Bar = 2 mm. **B**–**D** The average body weight of 3.rd-instar larvae, pupae, and adults was compared between the pirk-KO and WT groups. **E** The ratio of pupation in the pirk-KO and WT. **F** The survival curves and median survival (days) of the WT and *pirk* mutant demonstrate a significantly shortened lifespan. **G** The number of eggs laid per female with various crosses of the wild or pirk-KO line. Values presented represent the mean ± SEM with a sample size of *n* = 10 for measurements of body weight and pupation rate, *n* = 6 for fertility. The data were compared to that of WT flies using unpaired 2-tailed Student’s *t*-test (**B**–**D**), Welch’s *t*-test (**E**), log-rank (Mantel-Cox) test (**F**), and two-way RM ANOVA followed by Sidak’s multiple comparisons test (**G**). An asterisk indicates a significant difference from the control (*, *p* < 0.05; **, *p* < 0.01; ***, *p* < 0.001)

A series of physiological parameters were evaluated in the pirk-KO flies to ascertain the impact of the loss of *pirk* on fitness. A slight delay in larval growth was observed in pirk-KO individuals. The administration of a standard food diet ensured the progression of all pirk-KO strains through the larval, pupal, and adult stages, although their body size was marginally diminished in comparison to the WT individuals (Fig. 3A). The retardation of growth resulted in lighter larvae, pupae, and adult flies compared to the WT, with reductions in body weight of 14.31%, 13.64%, and 25.58%, respectively (see Fig. 3B–D). The pupation success rate of pirk-KO larvae was 87.67%, which is lower than that of WT (Fig. 3E), while the knockout of *pirk* did not affect the rate of adult emergence. A significant decrease in size was observed at different stages of ovarian development in comparison to the WT.

The lifespan of 50 pirk-KO and 50 WT fly pairs (each consisting of one female and one male) was examined. It was observed that the median lifespan of the WT controls was 27 days, while that of the pirk-KO flies was 20 days (Fig. 3F). This suggests that the lifespan of the WT flies was 25.92% longer than that of the mutants. This is evident in adult flies following *pirk* deletion, which may contribute to the shortened lifespan observed in *pirk* mutants.

The daily and total fecundity of each 15-pair cohort of flies was compared among the WT strain, the pirk-KO strain, pirk-KO females crossed with WT males (pirk-KO♀ × WT♂), and WT females crossed with pirk-KO males (WT♀ × pirk-KO♂). The cumulative number of eggs laid by mated females of the WT♀ × pirk-KO♂, pirk-KO♀ × WT♂, and pirk-KO strain during 24 days was significantly reduced by 18.86%, 39.63%, and 55.32%, respectively, in comparison to those of the WT group (Fig. 3G). It is noteworthy that females of the WT strain crossed with males of the pirk-KO strain produced a greater number of eggs than females of the pirk-KO strain mated with males of the WT strain, although this number was not as high as that produced by the WT strain. These findings indicate that the *pirk* knockout affects the reproductive capacity of flies, particularly the females, resulting in a reduction in fecundity. The collective findings indicated that the knockout of *pirk* impeded larval growth and reduced adult fecundity, likely due to excessive immunodepletion.

### Loss of *pirk* resulted in over-activated immunity

**Fig. 4:**
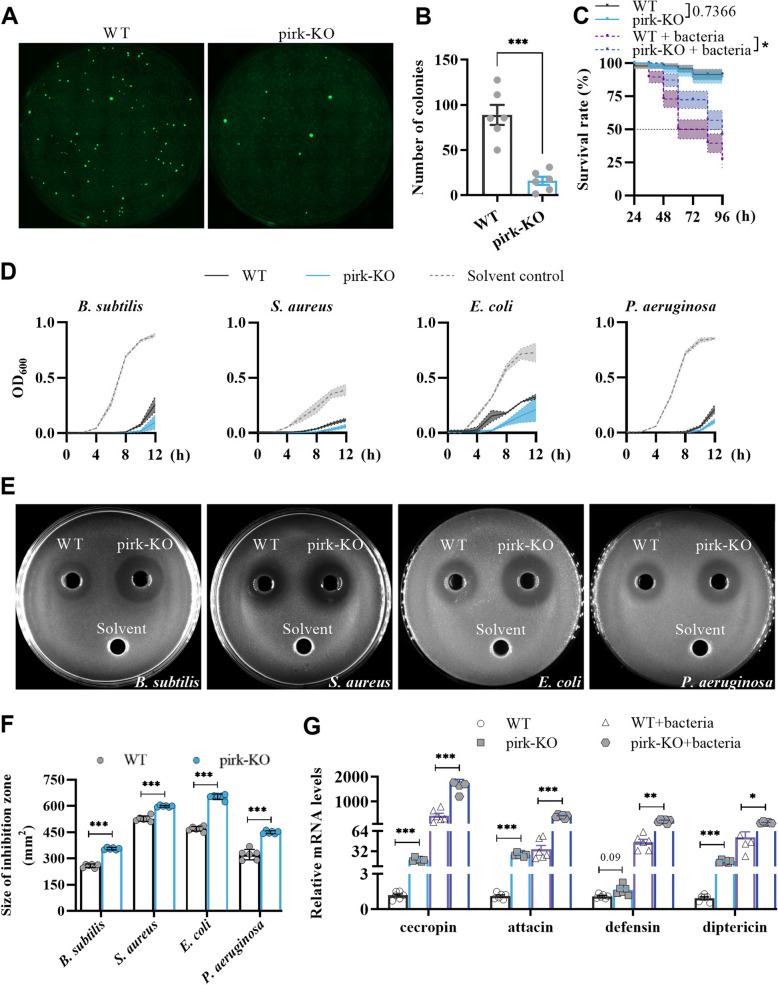
Loss of *pirk* resulted in immune overactivation and enhanced host resistance to bacterial infection. **A** Green fluorescent protein-expressing bacteria isolated from the hemolymph were observed under a fluorescence microscope. GFP-expressing *E. coli* (green) were injected into the larvae. One hour later, the larvae were punctured using forceps to extract the hemolymph, and then the hemolymph was plated onto resistant plates. After overnight incubation, the number of colonies was recorded. **B** Bacterial counts (CFU) were assessed in hemolymph samples collected from both pirk-KO and WT larvae 1 h following injection with GFP-expressing *E. coli* (*n* = 6, Student’s *t*-test). **C** Viability of pirk-KO and WT larvae after immune challenge with or without *S. marcescens* (*n* = 3 cohorts, total 150 larvae, log-rank (Mantel-Cox) test). **D**–**F** Antimicrobial activity evaluation of crude protein extracts from pirk-KO and WT larvae: growth curve dynamics and inhibition zones assay quantification (inhibition zone diameter) (*n* = 6, Student’s *t*-test). Solvents were used as negative controls. **G** The transcript levels of antimicrobial peptide genes in pirk-KO and WT adults following immune challenge with or without bacteria. The AMP genes and their respective IDs are as follows: *cecropin* (LOC101901181), *attacin* (LOC109612355), *defensin* (LOC101887872), *diptericin* (LOC101896897). The expression level of each gene was normalized to that of the uninfected WT control (*n* = 6, Brown-Forsythe ANOVA test, Dunnett’s test). The error bar represents the mean ± SEM of results performed at least three times. An asterisk indicates a significant difference from the control (*, *p* < 0.05; **, *p* < 0.01; ***, *p* < 0.001).* p* value means that it is not significant

To investigate the potential immune function of *M. domestica* Pirk in vivo, mortality and bacterial clearance of Pirk following bacterial infection were examined. For the bacterial clearance assays, the persistence of *E. coli* engineered to express green fluorescent protein (GFP-expressing *E. coli*) in house fly larvae was monitored by counting colony forming units (CFUs). The result showed a significant reduction in the number of *E. coli* clones recovered from pirk-KO larvae compared to the WT group (Fig. 4A and B). Subsequently, bioassays were conducted to identify any differences in susceptibility to bacterial infection by *Serratia marcescens* between the pirk-KO and WT larvae (Fig. 4C). The results demonstrated that the survival rate of pirk-KO larvae was nearly identical to that of WT controls in the absence of pathogen infection. However, *pirk* mutants exhibited a notable enhancement in survival following infection, with 88.2% surviving the initial 48 h, compared to only 74.5% of WT flies. The survival rate of *pirk* mutant larvae remained 18.9% higher than that of the control group until 96 h post-infection, indicating that the absence of *pirk* may have enhanced the flies’ capacity to withstand bacterial infection.

The bactericidal activity of protein extracts derived from pirk-KO larvae against *Bacillus subtilis*, *S. aureus*, *E. coli*, and *Pseudomonas aeruginosa* was evaluated by analyzing microbial growth curves (Fig. 4D) and measuring the size of inhibition zone (Fig. 4E, F). The inhibitory effect of the pirk-KO protein extract was found to be significantly greater than that of the same amount of WT control, indicating that the deletion of *pirk* resulted in enhanced antimicrobial peptide synthesis in larvae. Subsequently, we examined the influence of *pirk* loss on the expression of a range of AMP genes, including *cecropin*, *attacin*, *defensin*, and *diptericin*, in the context of bacterial infection. A qRT-PCR assay was conducted, which revealed that larvae lacking *pirk* exhibited a higher expression of immune genes than control group, irrespective of septic injury (Fig. 4G). This indicates that the absence of *pirk* led to an excessive activation of the immune system.

### Loss of *pirk* affects food intake, metabolite levels, and metabolic rate

**Fig. 5:**
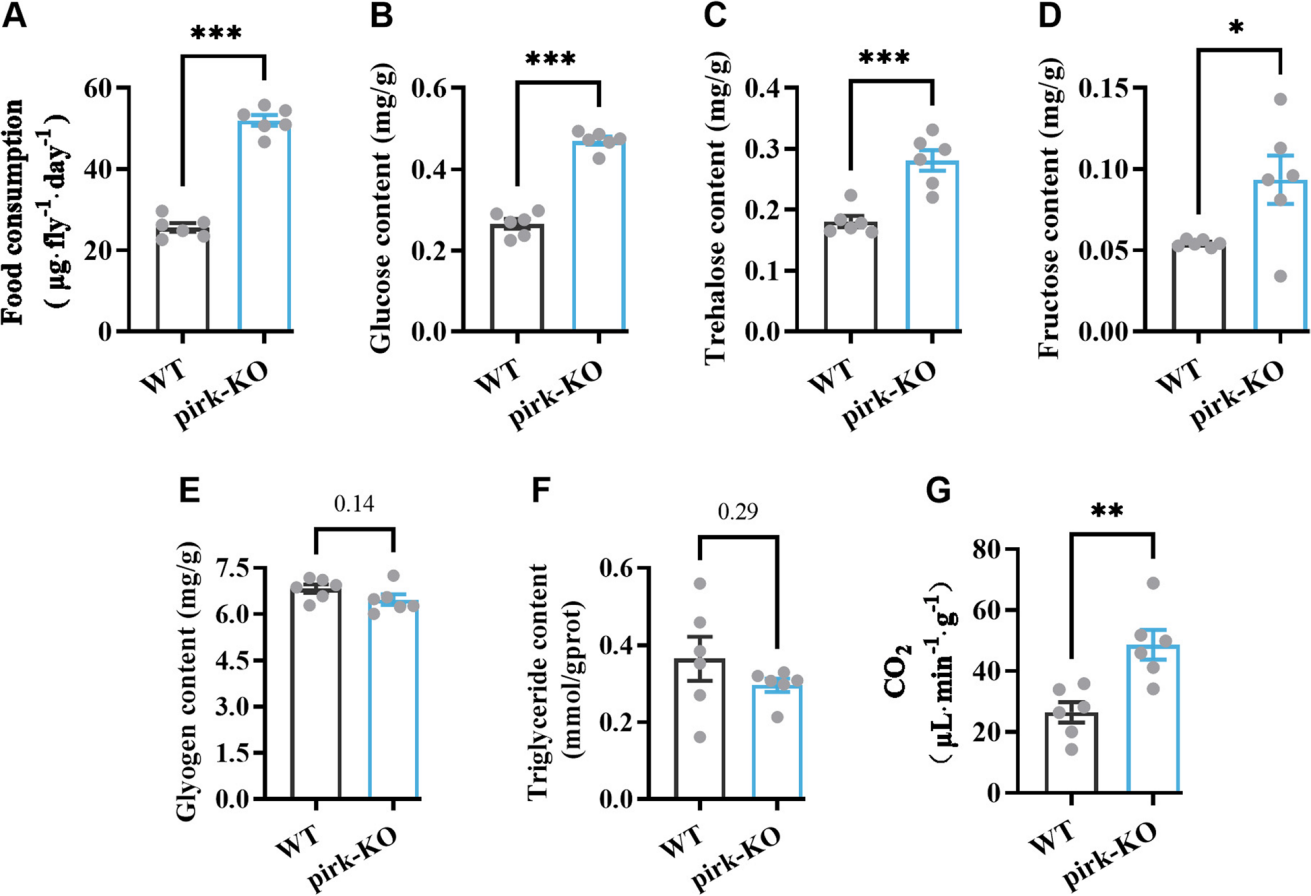
Measurements of food intake and metabolites in pirk-KO and WT house flies. **A** Measurements of blue label uptake after 30 min of feeding of house flies. **B**–**F** Contents of glucose, trehalose, fructose, glycogen, and triglyceride of pirk-KO and WT house flies. **G** CO_2_ production from pirk-KO and WT flies at 25 °C. Data are expressed as mean ± SEM of six independent experiments conducted as triplicates. *p < 0.05; **p < 0.01; ***p < 0.001; *p* value means that it is not significant. Statistical significance was assessed using unpaired 2-tailed Welch’s *t*-test (**A**, **D**, **F**) and Student’s *t*-test (**B**, **C**, **E**, **G**)

To determine the impact of Pick depletion on feeding behavior, 5-day-old flies were allowed to feed for 30 min on food items labeled with blue dye. Subsequently, the flies were sampled, and the quantity of ingested blue food was quantified using spectrophotometry. The results demonstrated that the pirk-KO flies exhibited a significantly elevated food intake in comparison to the WT flies, with a mean increase of approximately 101.69% (Fig. 5A).

The soluble sugar content in larvae was quantified, revealing that pirk-KO flies exhibited elevated levels of free glucose, trehalose, and fructose when normalized to protein content (as measured photometrically) in comparison to WT flies (Fig. 5B–D). Nevertheless, the glycogen and triglyceride contents in the pirk-KO flies were observed to be slightly reduced in comparison to the WT group (Fig. 5E and F).

To investigate the impact of *pirk* loss on metabolic rate, CO_2_ production from WT and pirk-KO flies was measured using the SSI respiratory metabolic system. Both newly eclosed pirk-KO and WT adults were raised at 25 °C for 5–7 days prior to measurement. At the experimental temperature of 25 °C, the amount of CO_2_ produced from *pirk* mutants was found to be 83.87% higher than that from WTs (Fig. 5G), indicating that the loss of *pirk* leads to an increased metabolic level.

### Transcriptome profiling highlights over-activated Imd signaling and reprogrammed metabolism in pirk-KO flies

To facilitate a comprehensive comparison of the differences in gene expression in response to bacterial infection between pirk-KO and WT flies, RNA sequencing was conducted on 5-day-old adult males post-challenge with a mixture of *S. aureus* and *E. coli*. This approach allowed for the examination of gene expression patterns in a controlled setting, providing insights into the molecular mechanisms underlying the observed differences in response to infection. Six hours post-infection, three replicate pools of pirk-KO and WT flies were collected and sequenced using the Illumina Novaseq platform. A total of 146.15 million and 156.22 million clean reads were sequenced from the two groups, respectively. Of these, an average of 65.86% and 59.32%, respectively, were mapped to the *M. domestica* genome using Hisat2. Furthermore, an optional de novo assembly step with StringTie was employed to assemble and quantify full-length transcripts representing novel genes, resulting in a total of 1213 novel transcripts.

**Fig. 6:**
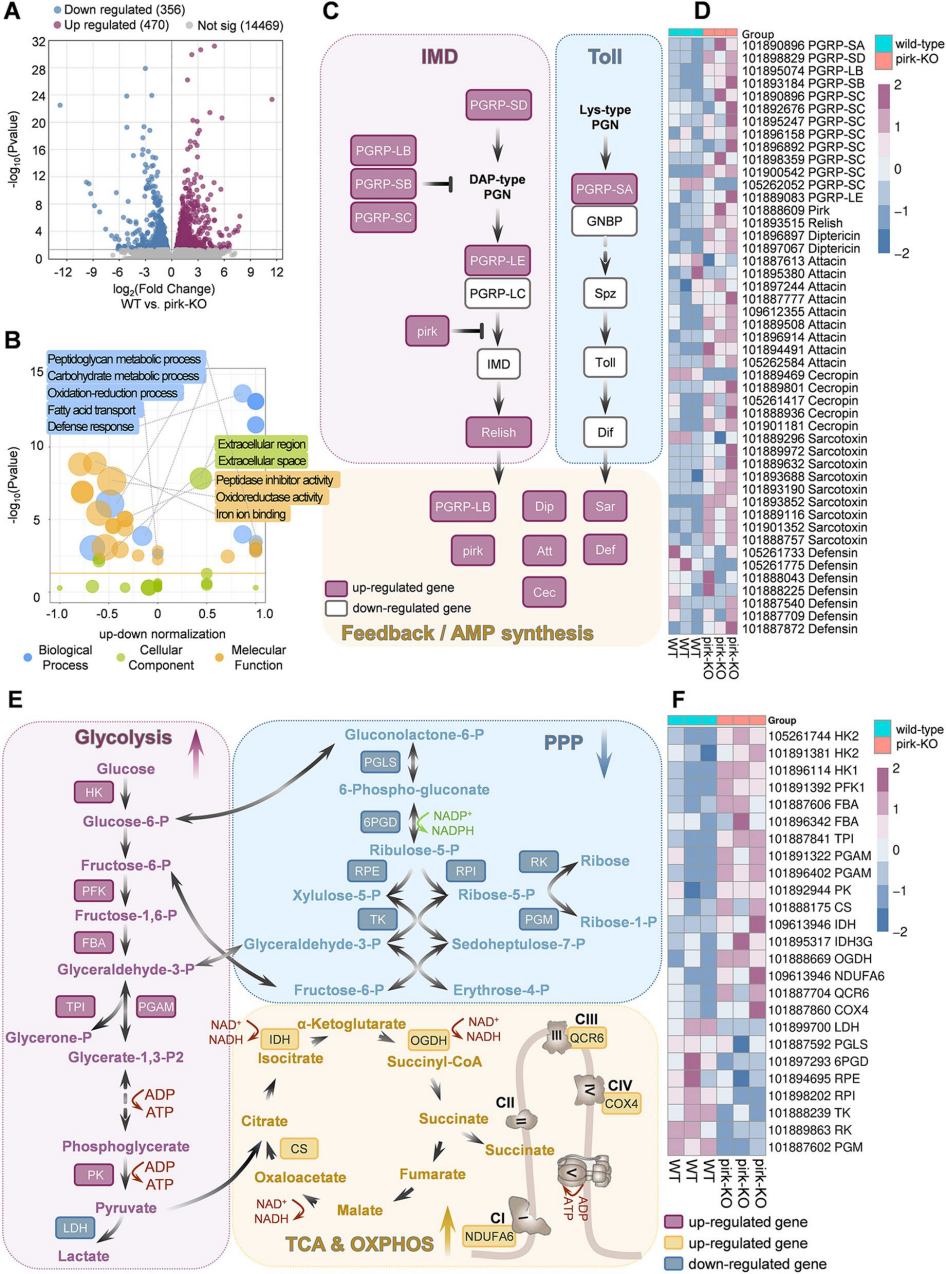
Bacterial infection on the transcriptome of adult flies between pirk-KO and WT strains. **A** Volcano plot highlighting differentially expressed genes in response to bacterial infection between pirk-KO and WT strains. **B** Gene ontology enrichment analysis of biological process, molecular function, and cellular component for up- and downregulated genes between pirk-KO and WT flies upon pathogen infection. **C** and **E** KEGG pathway map showing significant DEGs in Toll and Imd signaling and glycolysis/gluconeogenesis, pentose phosphate pathway (PPP), tricarboxylic acid (TCA) cycle, and oxidative phosphorylation (OXPHOS) between pirk-KO and WT strains following bacterial infection. **D** and **F** Heatmap over all samples showing the differentially expressed genes related to the Toll and Imd signaling pathway and central carbon metabolism pathway. Reference threshold: log_2_(fold-change) ≥ 1 or ≤ − 1; *p* value ≤ 0.05

A total of 826 DEGs were identified using the DESeq2 R package with the criteria of |log_2_FoldChange|≥ 1.0 and *p*adj ≤ 0.05. Of these, 470 were upregulated and 356 downregulated in pirk-KO compared to WT (Fig. 6A) (Additional file 2: SI-3). The GO enrichment analysis identified 31 significantly enriched biological processes of DEGs, which mainly included defense response and oxidation–reduction process, in addition to carbohydrate metabolism, peptidoglycan metabolism, fatty acid transport, and other processes. The majority of cellular components were found to be concentrated in the extracellular region. The enriched molecular functions included iron ion binding, oxidoreductase activity, peptidase inhibitor activity, and so forth, amounting to a total of 19 entries (Fig. 6B) (Additional file 2: SI-4). The KEGG pathway analysis revealed notable discrepancies in Toll and Imd signaling between the pirk-KO and WT strains (Fig. 6C). A comprehensive analysis demonstrated that the upregulated genes predominantly encompassed AMPs (e.g., *diptericin*, *attacin*, *cecropin*, *sarcotoxin*, and *defensin*), *PGRPs*, and *relish*, which were predominantly enriched in the Imd pathway as opposed to the Toll pathway (Fig. 6D) (Additional file 2: SI-5). Furthermore, there are several enriched but not significant (*p*adj > 0.05) pathways that warrant further investigation, including glycolysis/gluconeogenesis, glycine, serine and threonine metabolism, biosynthesis of amino acids, glutathione metabolism, glycerophospholipid metabolism, and fatty acid elongation. The DEGs involved in energy metabolism pathways, including glycolysis/gluconeogenesis (EMP), the pentose phosphate pathway (PPP), the tricarboxylic acid (TCA) cycle, and oxidative phosphorylation (OXPHOS), were manually screened and their expression levels were analyzed (Fig. 6E). The upregulated expression of pivotal genes involved in glycolysis (e.g., hexokinase (HK), 6-phosphofructokinase (PFK), fructose-bisphosphate aldolase (FBA), triosephosphate isomerase (TPI), phosphoglycerate mutase (PGAM), and pyruvate kinase (PK)) and the TCA cycle (e.g., the upregulation of citrate synthase (CS), isocitrate dehydrogenase (IDH), and 2-oxoglutarate dehydrogenase (OGDH)), along with OXPHOS (NADH dehydrogenase (NDUFA), ubiquinol-cytochrome c reductase subunit 6 (QCR6), and cytochrome c oxidase subunit 4 (COX4)), indicated an enhanced central carbon metabolism in pirk-KO. The reduced expression of L-lactate dehydrogenase (LDH) and in pirk-KO flies indicated that a greater proportion of the glycolytic product pyruvate was ultimately metabolized through the tricarboxylic acid cycle and oxidative phosphorylation pathway, thereby optimizing the production of ATP for cellular functions. The PPP is responsible for the generation of NADPH and ribose-5-phosphate (R5P), which are essential for maintaining cellular redox homeostasis and facilitating biosynthesis. Multiple important enzymes in the PPP, including 6-phosphogluconolactonase (PGLS), 6-phosphogluconate dehydrogenase (PGD), ribulose-phosphate 3-epimerase (RPE), transketolase (TK), ribose-5-phosphate isomerase (RPI), ribokinase (RK), and phosphoglucomutase (PGM), were downregulated in pirk-KO flies (Fig. 6F) (Additional file 2: SI-6). These findings suggest that *pirk*-KO flies undergo metabolic reprogramming characterized by a reduction in PPP flux and impaired anabolism. This shift appears to prioritize ATP production in response to excessive immune activation, rather than nutrient incorporation into biomass for cell proliferation.

### Imd pathway attenuation by Relish RNAi mitigates growth defects in pirk-KO house flies

Feeding assays demonstrated that dsRelish-treated groups exhibited a 40.98% reduction in *Relish* transcript levels and significant downregulation of Imd pathway-associated AMPs compared to dsGFP controls (Additional file 3: Fig. S1). Concomitantly, these groups showed enhanced physiological performance compared to dsGFP, including increased mean body weights of third-instar larvae (5.03%), pupae (5.43%), and adults (14.07%), along with elevated pupation rates (13.79%), lifespan of adult (2–3 days), and fecundity (5.70%) (Additional file 3: Fig. S2). Although these parameters remained suboptimal relative to wild-type levels, the findings substantiate the energy allocation hypothesis-suppression of Relish-mediated Imd pathway activation partially mitigates immune-induced growth trade-offs. The incomplete phenotypic rescue suggests *pirk* deficiency likely disrupts compensatory regulatory mechanisms essential for complete metabolic rebalancing.

## Discussion

Historically, infectious diseases have been the primary cause of mortality in humans [21]. The prevailing view among biologists is that natural selection favors individuals with ever-stronger immunity [1]. However, evolutionary ecologists have proposed that defensive responses can be energetically costly. It is hypothesized that upon infection by a pathogen, hosts are compelled to reallocate more resources and energy to immune defense, thereby reducing the allocation to normal life-history traits [22]. Defense-associated resource reallocation results in trade-offs between immunity and multiple biological processes, including host metabolism [5], growth [23], survival [24], and reproduction [25]. The fitness costs incurred by hosts during infection can be attributed to both direct pathogen exploitation and immune activation [25]. Nevertheless, it remains unclear which of pathogen exploitation and immune activation is the greater contributor to fitness costs. The majority of previous studies investigating the costs of infection were conducted under conditions of pathogen exposure [26, 27], and thus did not exclude the costs of pathogen exploitation. Therefore, the results do not accurately reflect the actual cost of immunity. The present study employed the pirk-KO house fly, a model of non-pathogenic immune activation, to investigate the costs associated with immune responses.

A novel gene exclusive to Insecta, designated *pirk*, exhibits rapid adaptation dynamics. A comprehensive search of the genome databases in GenBank (conducted Oct 12, 2024) revealed the presence of all 147 *pirk* homologous genes in 147 species, representing six distinct insect orders. The *M. domestica* Pirk protein exhibits 26.86% identity with the *D. melanogaster* Pirk protein, which represents the founding member of the Pirk family [15]. A Pirk member has recently been characterized in the mosquito *Aedes aegypti*, which shares only 29% similarity with its *D. melanogaster* ortholog [13]. Prior research has demonstrated that elevated levels of pirk expression inhibit immune system activation by negatively regulating Imd signaling during infection with Gram-negative bacteria. Additionally, Pirk itself is subject to regulation by the Imd pathway [15, 28]. And studies demonstrate that mutations in intracellular negative regulators of the Imd/NF-κB pathway predispose flies to toxic levels of AMPs, resulting in early locomotor defects and extensive neurodegeneration [29]. Hyperactivation of the Imd immune pathway in *Drosophila* disrupts gut microbial homeostasis, leading to dysbiosis, which is characterized by an increased abundance of specific bacterial taxa [30].

However, our observations indicated that *M. domestica pirk* can upregulate expression in response to both Gram-negative and Gram-positive bacteria, suggesting a versatile role in innate immunity. Given that Gram-negative and Gram-positive bacteria typically activate the Imd and Toll pathways [19, 20], this implies that Pirk may function at a point of crosstalk or convergence between these pathways, potentially contributing to broader immune regulation in house flies compared to *Drosophila*. Additionally, our findings revealed that *pirk* was predominantly expressed in the intestine and the fat body, which underscores its involvement in maintaining gut homeostasis and ensuring an appropriate level of systemic immunity.

The immunoregulatory function of Pirk as a negative regulator was further verified in a null mutant generated in this study. We found that deletion of *pirk* enhanced transcription of antibacterial peptide genes and humoral bacteriolytic activity in larvae in the presence or absence of bacterial infection. Consistent with this, the pirk-KO flies exhibited significantly enhanced resistance to infection by *S. marcescens* compared to the wild-type strain. Taken together, we speculate that Pirk may not only act as an inductive negative regulator to inhibit overactivation of the immune response and prevent immunopathology during infection, but also as a constitutive negative regulator to maintain appropriate immune levels in normal flies and prevent unnecessary energy expenditure.

*pirk*-deficient *M. domestica* provides an ideal model of autoimmune disorder with an unbridled Imd signaling and a sustained activated immune response, accompanied by growth and reproductive defects. In comparison to the WT house fly, the pirk-KO strain displays a reduction in the growth rate of larvae and a shortened lifespan of adults. It is hypothesized that there is a correlation between body size and lifespan and the functional costs of immune activation [31]. Furthermore, a deficiency in *pirk* has been associated with a decline in female fertility, as evidenced by the suppression of the vitellogenin gene expression, the emergence of ovarian dysplasia, and a reduction in egg production. Similarly, in the reproductive process of *A. aegypti* following a blood meal, 20-hydroxyecdysone (20E)-induced *pirk* regulates the trade-off between reproduction and immunity by inhibiting Imd, thereby maintaining optimal fertility [13]. The trade-off between immune defense and reproduction, two of the most energy-intensive physiological processes, has been repeatedly observed in a diversity of female insects [11, 32]. The reduction in reproductive output during infection is frequently attributed to an energy shortage resulting from direct pathogen exploitation as well as immune fueling. However, the cost of immune activation alone has not been the subject of rigorous evaluation.

Ordovás-Montañés et al. observed that following transient exposure to live and heat-killed *S. aureus*, the fecundity of *Caenorhabditis elegans* nematodes exhibited a similar short-term delay in offspring reproduction. The authors argue that immune upregulation can independently cause reproductive delay [25]. In the present study, we used the *pirk* mutant flies to exclude the interference of pathogens and demonstrate the negative effects of immune activation alone on the host. Our findings suggest that the unwarranted immune activation in the pirk-KO house flies results in the expenditure of excessive energy at the expense of growth and reproduction. The results of subsequent metabolite measurements and metabolic rate assays supported this view. The elevated “blood sugar” levels (glucose and trehalose) and diminished energy-storage substance contents (glycogen and triglyceride) suggest an augmented energy metabolism in the *pirk* mutants, a phenomenon corroborated by the observation of elevated CO_2_ production in the *pirk* mutants relative to the WTs. Glucose and trehalose are the primary blood sugars present in insect hemolymph, serving as an immediate source of energy and playing a pivotal role in growth and development [33]. Glycogen and lipids represent the primary energy storage forms in insects, with synthesis and breakdown processes subject to tight metabolic regulation [34]. Notably, *pirk* deficiency did not result in feeding inhibition but rather led to increased food intake in flies. An alternative viewpoint posits that the costs associated with immune responses are linked to alterations in resource acquisition, rather than resource reallocation. The reduction in resource acquisition resulting from “infection-induced anorexia” is identified as the primary driver of metabolic changes and fecundity costs associated with immune activation [5]. However, the results of the present study indicate that the observed compromised reproductive and growth traits in pirk-KO flies were due to a reallocation of resources, rather than a decline in resource acquisition. Indeed, in this case, pirk-KO flies were observed to mitigate the cost of immune activation by increasing their food intake.

Upon infection, the immune cells rapidly alter their metabolic profile in order to obtain sufficient energy to engage in defensive or homeostatic processes. The vital metabolic pathways with a direct connection to immune reprogramming include glycolysis, the tricarboxylic acid (TCA) cycle, oxidative phosphorylation (OXPHOS), the PPP, fatty acid oxidation (FAO), and fatty acid synthesis (FAS) [35]. In the present study, we employed RNA-sequencing analysis to investigate the global gene profile of pirk-KO flies during bacterial infection. The KEGG analysis corroborated a notable elevation in the Imd pathway in the pirk-KO flies in comparison to the WT individuals, which aligns with the observed upregulation of antimicrobial peptide expression and augmented bacteriolytic activity. The loss of *pirk* resulted in a modified central carbon metabolism (CCM), including glycolysis, the TCA cycle, and the PPP. CCM represents a significant source of energy for cell growth and development, while also providing precursors for other metabolic activities [36]. During glycolysis, glucose is broken down to form pyruvate, which in turn produces adenosine triphosphate (ATP). Pyruvate can then be metabolized in the mitochondria via the TCA cycle coupled with OXPHOS to produce ATP and CO_2_. The elevated expression of pivotal genes involved in glycolysis and the TCA cycle indicated an enhanced energy production, which is essential to support the high energy expenditure associated with hyperactivated immune defense in pirk-KO. CO_2_ production is typically regarded as an effective indicator of substrate oxidation and energy expenditure, offering insights into metabolic state. In this study, we employed the SSI respirometry system to detect a significantly higher rate of carbon dioxide synthesis in pirk-KO flies than in the wild type, indicating a stronger basal metabolic rate. In addition, the downregulation of multiple crucial enzymes in the PPP suggests an impaired anabolism, which may be a direct cause of growth inhibition and reduced fertility in Pirk-KO house flies.

## Conclusions

Pirk is a negative regulator of the house fly Imd pathway. Its deletion leads to unregulated immune activation and promotes energy-wasting phenotypes, including delayed larval growth and reduced fecundity in female flies. pirk-KO house flies provide an excellent model for the study of ecological immunology, which allows researchers to disregard the energy expenditure incurred by pathogens while focusing on the direct costs of immune activation. The immune responses are costly, and the costs are manifested in increased metabolic rates and trade-offs in life-history traits such as lifespan, growth, and reproduction.

## Methods

### Rearing of house flies

The wild-type (WT) *Musca domestica* strain used in this study is a laboratory-adapted population originally provided by Miss Fengqin He from the Institute of Zoology, Chinese Academy of Sciences. This population has been maintained under controlled conditions for over 15 years in our laboratory, where larvae were reared on a bran-based artificial medium at 25 °C. Adult flies were continuously mass-reared under strict hygiene protocols to minimize microbial contamination, with their diet consisting of powdered milk, sugar, and water. The adult rearing environment was maintained at 28 °C with 60–80% relative humidity and a 12 h/12 h (light/dark) photoperiod.

### Screening of *pirk* gene in *M. domestica*

A BLAST search was conducted against the *M. domestica* genome (GenBank accession No. GCA_000371365.1) and transcriptome data in our laboratory [19] using the amino acid sequence of Pirk from *D. melanogaster* as the query sequence. The cDNA sequence of the candidate *M. domestica pirk* was confirmed by sequencing subsequent to reverse transcription-polymerase chain reaction (RT-PCR) using gene-specific primers (Additional file 4: Table S1). To improve the identification of the *pirk* family, the researchers carried out a comprehensive search of the Nr database in GenBank and UniProt. The ClustalW program was employed to align the amino acid sequences of the Pirk homologues, and a maximum-likelihood phylogenetic tree was constructed through MEGA 6.0 software. The amino acid sequence of *M. domestica* Pirk was then subjected to analysis using the Expert Protein Analysis System (ExPASy, https://www.expasy.org).

### RNA extraction, cDNA synthesis, and qRT-PCR

RNA was extracted from 2nd-instar larval carcass (used in bacterial infection experiments) or 3rd-instar larvae (used for tissue dissection) using the RNAiso reagent (TaKaRa, China) in accordance with the manufacturer’s instructions. Single-stranded cDNA was synthesized from 2 μg of total RNA using the PrimeScript RT reagent Kit (TaKaRa, China) with gDNA Erase. The qRT-PCR was conducted on target genes in triplicate using a LightCycler system (Roche, USA) with the SYBR Green kit (TaKaRa, China). The primers utilized for qRT-PCR are presented in Table S1. The levels of expression of the target genes were determined by comparing the cycle threshold value (Ct) to that of the reference gene, *β-actin*. All samples were normalized to the ΔCt value of *β-actin* to derive a ΔΔCt value (ΔCt target − ΔCt reference). The final relative expression was calculated using the following formula: *F* = 2^−ΔΔCt^ [37].

### Establishment of *pirk* mutants using CRISPR/Cas9

The gene sequence for *M. domestica pirk* (no. XM_005188521.4) was obtained from GenBank, and the guide RNA (gRNA) target site was selected from the second exon. The gRNA sequence comprised 20 nucleotides, with a protospacer adjacent motif (PAM) located at the 3′ end. To evaluate the potential for off-target efficacy, the sequence was subjected to analysis using Cas-OFFinder (http://www.rgenome.net/cas-offinder/) in comparison to the *M. domestica* genome. The gRNA was synthesized using the GeneArt Precision gRNA Synthesis Kit (Invitrogen, USA). The purified gRNA and Cas9 protein (Invitrogen, USA) were combined to achieve final concentrations of 200 and 300 ng/μL, respectively. Subsequently, the solution was microinjected into freshly laid eggs within 20 min using an IM-300 microinjector (Narishige, Japan) and a needle produced from a micropipette puller (Sutter Instrument, USA). The embryos were subsequently cultivated in an incubator maintained at 28 °C. The surviving larvae were promptly transferred to a growth chamber and reared in accordance with the aforementioned protocol. Subsequently, the surviving G0 adults were individually backcrossed with wild-type (WT) flies, resulting in the generation of G1. Genomic DNA was extracted from the hind leg of each G1 adult using the TIANamp Genomic DNA Kit (TIANGEN, China) and employed as a template for PCR. PCR was conducted using the primers outlined in Table S1 to amplify DNA fragments encompassing the target site. A control experiment was conducted using a fragment amplified from WT DNA. The mutations were subsequently analyzed using polyacrylamide gel electrophoresis (PAGE) and Sanger sequencing. G1 heterozygotes, which carried the same mutation, were then self-crossed to obtain homozygous G2 mutants.

### Observation of growth and lifespan of the pirk-KO *M. domestica*

Eggs laid by female WT and pirk-KO flies were cultured in bran medium; 3rd-instar larvae were collected, boiled briefly for 30 s and then preserved in 80% ethanol. The straightened larvae and pupa were photographed using a microscope (SMZ1500; Nikon, Japan). Biometric measurements of the body weight of larvae, pupae, and adult flies were taken; approximately 100 larvae, 100 pupae, and 100 adult flies were divided into ten groups, each group being considered a replicate, using an analytical balance manufactured by Sartorius in Germany. Furthermore, the duration of pupation success rates was recorded. To assess the lifespan of the flies; 100 newly eclosed individuals (50 females and 50 males) were collected from each line. The flies were maintained at 28 °C under a 12:12 light–dark cycle and provided with a diet of 10% milk water. The number of surviving individuals in each group was counted on a daily basis until all individuals had died. This process was repeated five times, with a new cohort of flies being collected each time. The experiment aimed to assess the impact of the *pirk* knockout on reproductive capacity by analyzing the daily and total fecundity of flies across four groups: WT strain, pirk-KO strain, pirk-KO females crossed with WT males (pirk-KO♀ × WT♂), and WT females crossed with pirk-KO males (WT♀ × pirk-KO♂). In each group, 15 cohorts of flies were paired, and the cumulative number of eggs laid by mated females was measured over a 24-day period (*n* = 6). The fecundity results were then compared across the groups to evaluate differences and determine the effect of the *pirk* knockout.

### Analysis of bacterial clearance by loss of *pirk*

The bacterial clearance assay was conducted as previously described [38]. Briefly, GFP-expressing *E. coli* were cultured in LB medium containing ampicillin. Bacteria in the logarithmic growth phase were collected by centrifugation at 6000 g for 5 min, washed three times with TBS (20 mM Tris, 150 mM NaCl, pH 8.0), and resuspended in TBS to an OD_600_ of 1.0. A volume of 0.1 μL of bacterial suspension was microinjected into WT or pirk-KO larvae using the Nanoliter2020 (WPI, USA). After 30 min, larvae were washed, dried, and pierced with forceps to collect hemolymph, which was mixed with 200 μL of cold anticoagulant solution (40 mM NaCl, 27 mM trisodium citrate dihydrate, 100 mM glucose). Once 80 μL of hemolymph was obtained, 50 μL of it was diluted and plated onto resistant plates. Colony counts were recorded after overnight incubation.

### Bacterial challenge and survival statistics

To ascertain the potential involvement of *M. domestica pirk* in the resistance to bacterial infection, septic injury experiments were conducted on 5-day-old male flies of the pirk-KO and WT strains. The thoraces of the flies were punctured with a 0.1-mm dissecting needle that had been immersed in a concentrated mid-log phase culture of bacteria (*Serratia marcescens* NVIT01 and *Staphylococcus aureus* ATCC 25923) at an OD of 1.0. Each treatment group consisted of 100 individuals and was replicated three times. The flies were incubated at a temperature of 25 °C, and the number of surviving individuals in each group was recorded daily for 4 days. The survival percentage was calculated as the number of survivors divided by the number of initial individuals. At 48 h post-bacterial challenge, total RNA was extracted from various strains, with six flies pooled per RNA sample, and used for the analysis of AMP genes expression via the qRT-PCR method.

### Evaluation of the antibacterial activity of crude protein extracts from pirk-KO larvae

Whole pirk-KO or WT larvae were employed for the extraction of antimicrobial compounds. The 3rd larvae were subjected to thorough homogenization in a solution (90% methanol, 1% glacial acetic acid, 9% deionized water) (volume percent). Solvent was used as a parallel control sample during the extraction process. Following rotary evaporation of the supernatant, the concentrate obtained is dissolved in 0.1% trifluoroacetic acid. An equal volume of n-hexane is then added and mixed thoroughly. Centrifuge the mixture at 12,000 rpm for 10 min and collect the middle and lower aqueous phases. Add an equal volume of ethyl acetate to the aqueous phase and centrifuge at 12,000 rpm for 10 min. Collect the lower aqueous phase, evaporate to dryness on a rotary evaporator, and redissolve in sterile water.

The crude extract of the same larval weight was then tested for antibacterial activity against *Bacillus subtilis* ATCC 6633, *S. aureus* ATCC 25923, *Escherichia coli* ATCC 25922, and *Pseudomonas aeruginosa* ATCC 27853. These bacteria were cultured in LB medium at 37 °C overnight and subsequently collected by centrifugation at 6000 g for 5 min. The bacteria were then transferred to fresh Poor’s broth medium, comprising 1% tryptone, 0.5% NaCl, and a pH of 7.5, after rinsing with PBS. Subsequently, 15 μL of the crude protein extract or solvent control was added to a 96-well culture plate containing 135 μL of the bacterial solution (OD_600_ = 0.2, 100 dilution). The plate was incubated for 12 h at 23 °C, and the absorbance at 600 nm was measured at 4-h intervals using a Synergy HTX multifunctional microplate reader (BioTek, USA) to assess bacterial concentration. This process was repeated on three occasions.

Single colonies were picked and mixed in LB liquid medium and the suspension was prepared by shaking at 37 °C overnight. *B. subtilis*, *S. aureus*, *E. coli*, and *P. aeruginosa* were plated on LB culture plates. One hundred fifty microliters of crude extract samples were then added to the culture plates and incubated at 37 °C in an inverted culture for 12 h. Solvent was used as a parallel control. After incubation, the inhibition zones were recorded. The size of the inhibition zones was rigorously measured using ImageJ software. All experiments were performed in triplicate.

### Quantification of food intake in flies

The quantity of food consumed was quantified by labeling the food with a colorimetric dye, as previously described with minor modifications [39]. Ten 5-day-old WT or pirk-KO flies (5 females and 5 males) were randomly selected and fed a 10% milk solution containing 5 mg/mL bromophenol blue for 24 h after 12 h of starvation. Subsequently, the flies’ heads were removed with a scalpel, and their bodies were homogenized on ice in 500 μL of ice-cold TE buffer. The resulting homogenate samples were then subjected to centrifugation at 13,000 rpm for 10 min at 4 °C. The collected supernatant was subsequently subjected to another round of centrifugation under the same conditions. The quantity of blue pigment present in 100 μL of transparent supernatant was determined in 96-well plates using a Synergy HTX multifunctional microplate reader (BioTek, USA) to record the absorbance at 520 nm. To convert the absorbance values to the amount of food consumed per fly, a calibration relationship was established by measuring the absorbance of serial dilutions of a known food mass. The relative value obtained indicated the food intake of the flies. Six parallel sets were established for each group.

### Metabolite profiling

To investigate the effects of *pirk* depletion on metabolism, a series of measurements were conducted on a range of metabolites, including glucose, trehalose, fructose, glycogen, and triacylglycerol (TG), in both the pirk-KO and WT strains. First, four 5-day-old WT or pirk-KO flies (2 females and 2 males) were randomly selected and weighed using an analytical balance. The flies were homogenized in cold PBS containing 0.1% Triton X-100. To determine glycogen content, samples should be boiled for 10 min and cooled on ice for 3 min. Subsequently, centrifugation at 12,000 g for 10 min at 4 °C was performed, after which the supernatant was carefully collected and stored on ice for further analysis. The total protein concentration was determined using the BCA assay. The glycogen and TG contents were quantified using detection kits (Nanjing Jiancheng Bioengineering Institute, China) in accordance with the manufacturer’s instructions. Soluble sugars, including glucose, fructose, and trehalose, were quantified by high-performance liquid chromatography (HPLC) according to a method previously described by Min et al. [40], with minor modifications. In brief, the clear supernatant was mixed with an equal volume of acetonitrile, filtered through a 0.2-μm filter membrane, and then injected into the HPLC apparatus (Shimadzu, Japan), which was equipped with a pulseless pump (LC-20AD, Shimadzu, Japan) with a flow rate of 0.3 mL/min and a refractive index detector (RID-10A, Shimadzu, Japan). The separation of soluble sugars was conducted using a Carbomix H-NP10 column (7.8 × 300 mm) with a mobile phase comprising a 75/25 acetonitrile/water mixture. The soluble sugars present in the samples were identified and quantified by comparing the retention time and area of each peak of the HPLC profiles with those of the standard samples. Glucose, fructose, sucrose, and trehalose were obtained from Solarbio Life Science (Beijing, China). The concentration of all these metabolites was normalized to the protein level. These biochemical experiments were repeated three times from each batch.

### Measurement of metabolic rate in flies using a respirometry system

In insects, the input of oxygen (O_2_) is directly correlated with the output of carbon dioxide (CO_2_) and serves as a reflection of the level of metabolism. To ascertain whether the absence of *pirk* affects the host’s metabolic rate, the CO_2_ fluxes of pirk-KO and WT flies were monitored using a flow-through respirometry apparatus (Sable Systems International (SSI), USA). Flies of a specified age (5–7 days) were randomly selected and subjected to a 12-h starvation period. Following anesthesia, the flies were weighed on an analytical balance and then placed in breathing chambers, with 30 flies (15 females and 15 males) in each chamber. The airflow rate was set at 150 mL/min. Following a 10-min period of rest for the flies in the chambers at 25 °C, the CO_2_ output of each chamber was measured and recorded. The data were captured for 40 min and compared with the control (empty). The experiments were conducted in triplicate for each setup.

### RNA-seq analysis

A transcriptomic profiling of male pirk-KO and WT flies was conducted, utilizing three independent biological replicates for each strain. The 5-day-old adult pirk-KO and WT male flies were infected via puncture of the thorax with a thin needle soaked in a concentrated overnight culture of *E. coli* and *S. aureus*. At 12 h post-infection, total RNA was extracted from the flies using RNAiso reagent (TaKaRa, China). The quality of the extracted total RNA was evaluated using an Agilent 2100 bioanalyzer (Applied Biosystems, USA). The NEBNext mRNA Library Prep Reagent Set for Illumina was utilized for the preparation of paired-end libraries, which were subsequently sequenced in a paired-end format on an Illumina Novaseq platform (Illumina, Inc., USA) at Novogene, China. The 150-base pair paired-end reads were employed for subsequent analysis. Hisat2 version 2.0.5 was utilized for alignment of the raw reads to the *M. domestica* reference genome (GCF_000371365.1). The mapped reads of each sample were assembled using the StringTie software (version 1.3.3b) in a reference-based approach. The StringTie algorithm employs a novel network flow methodology and an optional de novo assembly step to assemble and quantify full-length transcripts representing multiple splice variants for each gene locus. The software program FeatureCounts, version 1.5.0-p3, was employed to enumerate the reads that were mapped to each gene. Subsequently, the fragments per kilobase of transcript per million mapped reads (FPKM) was calculated for each gene, with consideration given to the gene’s length and the number of reads mapped to it. The DESeq2 R package (1.20.0) was utilized for the purpose of differential expression analysis between the two strains. The resulting *p* values were adjusted according to the Benjamini and Hochberg method to control the false discovery rate. An adjusted *p* value of 0.05 and an absolute fold change of 2 were established as the threshold for significant differential expression. Gene Ontology (GO) and Kyoto Encyclopedia of Genes and Genomes (KEGG) enrichment analysis of the differentially expressed genes (DEGs) was performed using the clusterProfiler R package. The GO and KEGG terms were deemed to be significantly enriched by the DEGs if they exhibited a corrected *p* value of less than 0.05. The clusterProfiler R package was employed to examine the statistical enrichment of the DEGs in KEGG pathways. Subsequently, a heatmap was plotted by https://www.bioinformatics.com.cn, an online platform for data analysis and visualization.

### RNA interference (RNAi) of Relish in pirk-KO house flies

The DNA fragment encoding Relish (XM_005178615.4) was amplified by PCR using Relish-F/R primer pairs to generate the double-stranded RNA (dsRNA) expression construct. A green fluorescent protein (GFP) gene fragment derived from pEGFP-N1 (GenBank: U55762) served as the experimental control. Primers containing *Xho* I and *Kpn* I restriction sites were designed based on the GFP plasmid sequence for amplification. The amplified fragments were restriction-digested and cloned into the corresponding sites of the L4440 vector. The resulting constructs, designated dsRelish and dsGFP, were transformed into RNase III-deficient *E. coli* HT115. To produce dsRNA, the HT115 transformants were cultured overnight at 37 °C in LB medium supplemented with ampicillin (100 μg/mL) and tetracycline (12.5 μg/mL). The bacterial cultures were then diluted 100-fold in fresh medium and incubated until an OD_600_ of 0.5 was reached. dsRNA expression was induced with 0.4 mM IPTG for 4 h at 37 °C.

RNAi was performed by feeding pirk-KO larvae bacteria expressing dsRNA according to a previously described method [41]. Briefly, the bacteria were harvested by centrifugation at 10,000 g for 2 min, then resuspended in sterile water for larval feeding. Feeding experiments commenced at the 2nd-instar larval stage and continued until pupation, with the diet replaced daily. To evaluate RNAi efficiency and AMP gene expression levels (*cecropin*, *attacin*, *defensin*, and *diptericin*), RNA extraction and subsequent qRT-PCR analyses were performed on the 3rd-instar larvae using primers listed in Table S1. The evaluation of the phenotypic alterations induced by Relish knockdown in pirk-KO house flies was conducted by monitoring developmental parameters, including body weight, pupation rate, adult longevity, and fecundity. All measurements were conducted using standardized methodologies as previously described.

### Statistical analysis

Statistical analysis was performed using GraphPad Prism 9.0. Sample distribution was determined using the Shapiro–Wilk normality test. For parametric data, the 2-tailed unpaired Student’s or Welch’s *t*-test was used to determine differences between 2 groups. One- or 2-way ANOVA and the Sidak’s or Tukey’s test were used to evaluate > 2 groups. For nonparametric data, the Brown-Forsythe and Welch ANOVA test with the exact method was used to identify differences between 2 groups. Statistically significant differences between survival curves were determined by log-rank test. *p* values were 2-tailed and values < 0.05 were considered statistically significant. Data are presented as the mean ± SEM.

## Supplementary Information

Additional file 1: SI-1, SI-2. SI-1 147 Pirk sequences. SI-2 Multiple sequence alignments of the Pirks.

Additional file 2: SI-3, SI-4, SI-5, SI-6. SI-3 All up- and downregulated differentially expressed genes in Fig. 6A. SI-4 The top 10 ranked up- and downregulated differentially expressed genes in GO terms related to biological process (BP), cellular component (CC), and molecular function (MF) in Fig. 6B. SI-5 and SI-6 The heatmap data of gene expression in Fig. 6D and F.

Additional file 3: Figures S1 and S2. Fig. S1 qRT-PCR analysis of Relish knockdown efficiency and antimicrobial peptide transcript levels in pirk-KO mutants. (A) qRT-PCR was performed to assess Relish knockdown efficiency in pirk-KO mutants fed with dsRNA-expressing *E. coli* HT115. (B) Transcriptional levels of AMPs were analyzed to evaluate immune pathway modulation. Fig. S2 *Relish* knockdown rescues developmental abnormalities in pirk-KO house flies. (A) Comparative growth of larval, pupal, and ovarian development. (B–D) The body weight of 3rd-instar larvae, pupae, and adults. (E) Pupation rate comparisons. (F) Adult survival curves (log-rank (Mantel-Cox)). (G) Fecundity quantification across genotypes (two-way ANOVA). Values presented represent the mean ± SEM with a sample size of *n* = 10 for measurements of body weight and pupation rate, *n* = 3 for fertility. The data were used Brown-Forsythe and Welch ANOVA test (B and C), Kruskal–Wallis test (D and E). An asterisk indicates a significant difference from the control (*, *p* < 0.05; **, *p* < 0.01; ***, *p* < 0.001).

Additional file 4: Table S1. Table S1 PCR primers used in this study.

Additional file 5: The individual data values for Figs. 1C–E, 3B–G, 4B–G, 5A–G, S1, and S2.

## Data Availability

All data generated or analyzed during this study are included in this published article and its supplementary information files. The remaining raw project data (RNA-seq read data) is free for download on publication from NCBI Sequence Read Archive (SRA) under accession number PRJNA1252960 at: https://www.ncbi.nlm.nih.gov/sra/PRJNA1252960.

## Abbreviations

pirk: Poor Imd response upon knock-in
AMP: Antimicrobial peptide
WT: Wild type
gRNA: Guide RNA
PAM: Protospacer adjacent motif
RNAi: RNA interference
