# Supplementary figures and images for: Resource reallocation under persistent immune activation drives trade-offs between life history and immunity in pirk-deficient Musca domestica
Source: BMC Biol. 2025 Jul 22;23:220. doi: 10.1186/s12915-025-02324-6 (PMC12285093; doi:10.1186/s12915-025-02324-6)

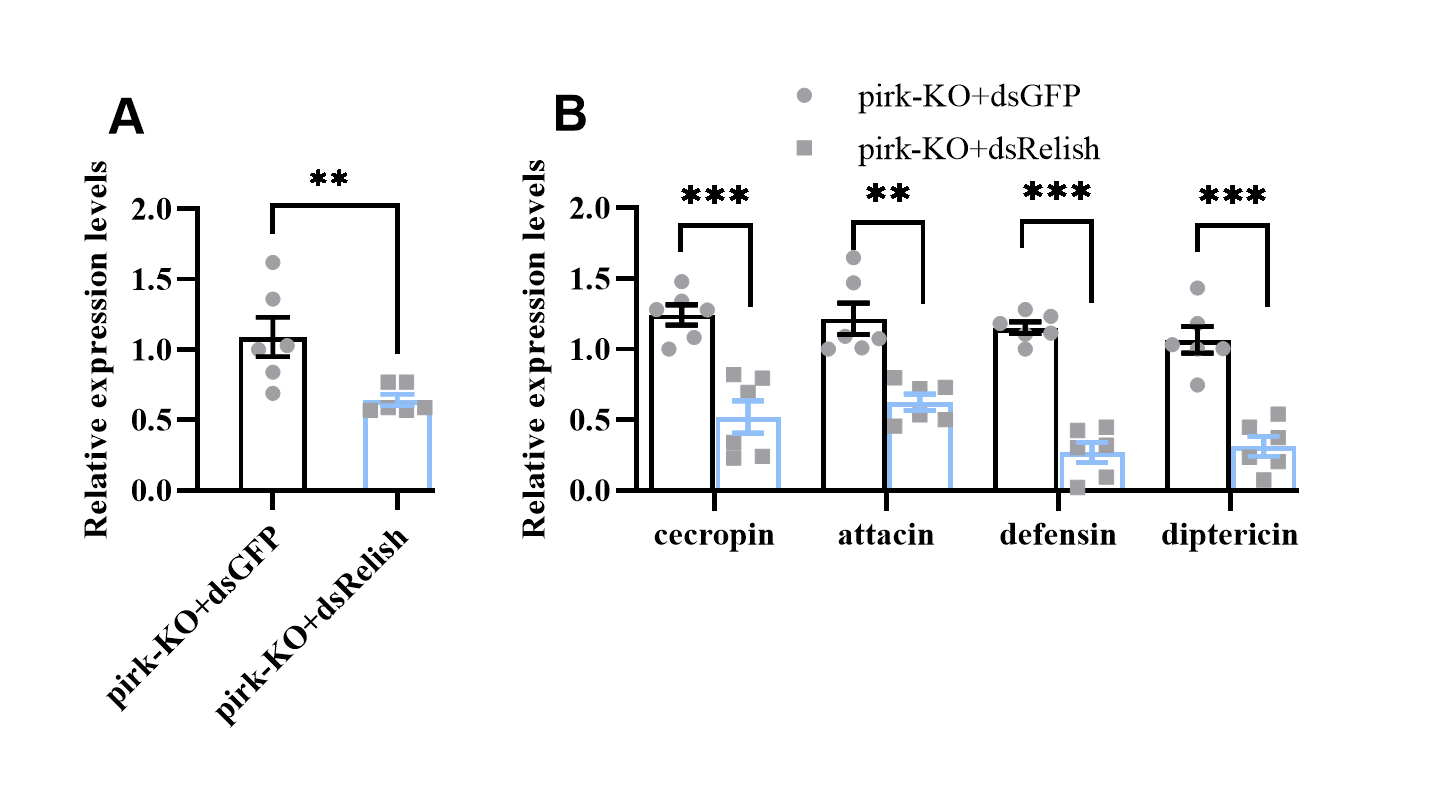

Supplement: Supplementary file 3 — Additional file 3: Figures S1 and S2. Fig. S1 qRT-PCR analysis of Relish knockdown efficiency and antimicrobial peptide transcript levels in pirk-KO mutants. (A) qRT-PCR was performed to assess Relish knockdown efficiency in pirk-KO mutants fed with dsRNA-expressing E. coli HT115. (B) Transcriptional levels of AMPs were analyzed to evaluate immune pathway modulation. Fig. S2 Relish knockdown rescues developmental abnormalities in pirk-KO house flies. (A) Comparative growth of larval, pupal, and ovarian development. (B–D) The body weight of 3rd-instar larvae, pupae, and adults. (E) Pupation rate comparisons. (F) Adult survival curves (log-rank (Mantel-Cox)). (G) Fecundity quantification across genotypes (two-way ANOVA). Values presented represent the mean ± SEM with a sample size of n = 10 for measurements of body weight and pupation rate, n = 3 for fertility. The data were used Brown-Forsythe and Welch ANOVA test (B and C), Kruskal–Wallis test (D and E). An asterisk indicates a significant difference from the control (*, p < 0.05; **, p < 0.01; ***, p < 0.001). [file 12915_2025_2324_MOESM3_ESM.zip › Additional file 3 FigureS1.tif]

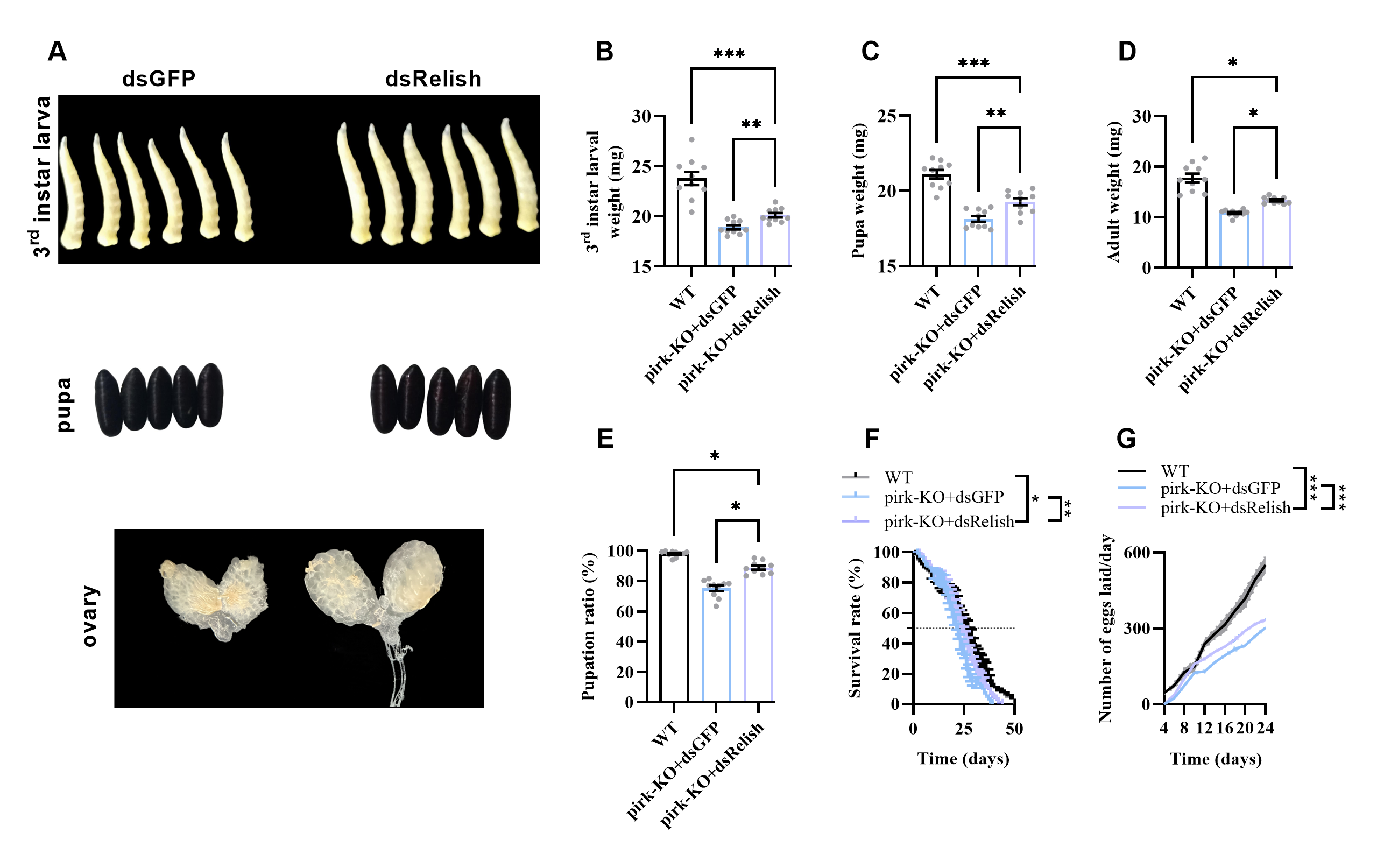

Supplement: Supplementary file 3 — Additional file 3: Figures S1 and S2. Fig. S1 qRT-PCR analysis of Relish knockdown efficiency and antimicrobial peptide transcript levels in pirk-KO mutants. (A) qRT-PCR was performed to assess Relish knockdown efficiency in pirk-KO mutants fed with dsRNA-expressing E. coli HT115. (B) Transcriptional levels of AMPs were analyzed to evaluate immune pathway modulation. Fig. S2 Relish knockdown rescues developmental abnormalities in pirk-KO house flies. (A) Comparative growth of larval, pupal, and ovarian development. (B–D) The body weight of 3rd-instar larvae, pupae, and adults. (E) Pupation rate comparisons. (F) Adult survival curves (log-rank (Mantel-Cox)). (G) Fecundity quantification across genotypes (two-way ANOVA). Values presented represent the mean ± SEM with a sample size of n = 10 for measurements of body weight and pupation rate, n = 3 for fertility. The data were used Brown-Forsythe and Welch ANOVA test (B and C), Kruskal–Wallis test (D and E). An asterisk indicates a significant difference from the control (*, p < 0.05; **, p < 0.01; ***, p < 0.001). [file 12915_2025_2324_MOESM3_ESM.zip › Additional file 3 FigureS2.tif]
